# Comprehensive characterization of adipogenesis-related genes in colorectal cancer for clinical significance and immunogenomic landscape analyses

**DOI:** 10.1186/s12944-023-01942-9

**Published:** 2023-12-07

**Authors:** Jing Han, Shangshang Li, Qiong Zhan, Yuchao Hu, Chaoxiang Zhong, Jie Yang, Zhengcai Qiu

**Affiliations:** Department of General Surgery, Shuyang Hospital of TCM, Suqian, Jiangsu Province China

## Abstract

**Objective:**

Colorectal cancer (CRC) is a major global health concern, necessitating the identification of biomarkers and molecular subtypes for improved clinical management. This study aims to evaluate the clinical value of adipogenesis-related genes and molecular subtypes in CRC.

**Methods:**

A comprehensive analysis of adipogenesis-related genes in CRC was performed using publicly available datasets (TCGA and GEO database) and bioinformatics tools. Unsupervised cluster analysis was employed to identify the molecular subtypes of CRC, while LASSO regression analysis was utilized to develop a risk prognostic model. The immunogenomic patterns and immunotherapy analysis were used to predict patient response to immunotherapy. Furthermore, qPCR analysis was conducted to confirm the expression of the identified key genes in vitro.

**Results:**

Through the analysis of RNAseq data from normal and tumor tissues, we identified 50 differentially expressed genes. Unsupervised cluster analysis identified two subtypes (Cluster A and Cluster B) with significantly different survival outcomes. Cluster A and B displayed differential immune cell compositions and enrichment in specific biological pathways, providing insights into potential therapeutic targets. A risk-scoring model was developed using five ARGs, which successfully classified patients into high and low-risk groups, showing distinct survival outcomes. The model was validated and showed robust predictive performance. High-risk patients exhibited altered immune cell proportions and gene expression patterns compared to low-risk patients. In qPCR validation, four out of the five key genes were consistent with the results of bioinformatics analysis.

**Conclusion:**

Overall, the findings of our investigation offer valuable understanding regarding the clinical relevance of ARGs and molecular subtypes in CRC, laying the groundwork for improved precision medicine applications and personalized treatment modalities.

**Supplementary Information:**

The online version contains supplementary material available at 10.1186/s12944-023-01942-9.

## Introduction

With a steadily growing occurrence and significant impact on mortality, colorectal cancer (CRC) is recognized as one of the leading malignant neoplasms worldwide. Despite considerable advancements in the timely detection and management of CRC, the underlying mechanisms that give rise to this disease remain complex and not entirely understood [1, 2]. Fat tissues are widely present in the body, serving not only as an energy storage site but also playing crucial roles in metabolic regulation, immune response, and hormone signaling. Therefore, the regulatory mechanisms of adipocyte differentiation in tumor development, known as adipogenesis, have received considerable research attention [3, 4]. Studies have revealed dysregulation of key adipogenic factors in breast cancer [5], prostate cancer [6], ovarian cancer [7], pancreatic cancer [8], and their altered expression has been associated with tumor progression, metastasis, and resistance to therapy. Targeting these genes or their downstream pathways has shown promise in inhibiting these cancer growths and improving treatment outcomes [9].

In recent years, bioinformatics technology has demonstrated great potential in revealing the molecular mechanisms of tumors [10–13]. By integrating high-throughput sequencing data and systems biology approaches, we can comprehensively understand the expression and regulatory patterns of Adipogenesis-Related Genes (ARGs), which are genes associated with adipocyte differentiation, in CRC. Given their probable significance in tumor development, advancement, and resistance to treatment, exploring these ARGs in-depth becomes imperative to elucidate the molecular intricacies of CRC and design tailored therapeutic strategies.

This article seeks to delve into the functions and potential mechanisms of ARGs in CRC by employing systems biology and bioinformatics analysis, and providing new insights and theoretical foundations for the pathological physiology research and development of treatment methods for CRC. Through a deeper understanding of the regulatory network of adipocyte differentiation in the occurrence and development of CRC, we hope to open up new possibilities for personalized and precise clinical treatment, thereby providing more effective treatment options for patients and reducing the incidence and related mortality rates of CRC.

## Materials and methods

### Data sets and preprocessing

The study flow chart is shown in Figure S1. For the retrieval of mRNA expression profiles and clinical data related to human colorectal cancer, a systematic computer search was performed on the Gene Expression Omnibus (GEO) dataset. One publicly accessible colorectal cancer cohort was retrieved from the GEO database. Additionally, we compiled a CRC cohort encompassing mutation and somatic copy number alteration data from The Cancer Genome Atlas (TCGA). After standardizing the raw count data through the R package ‘DEseq2’ [14], we further processed it to obtain transcripts per kilobase million (TPM) values using the R package limma [15]. The Affymetrix-generated original CEL files were sourced from either the GEO dataset or the ArrayExpress dataset. Thereafter, we conducted individual processing and normalization of the raw CEL files for each cohort, employing the R package affy [16]. Mapping of probes to gene symbols was performed with reference to the corresponding platform annotation file. When multiple probes were associated with the same gene symbol, the probe displaying the most sensitive signal was selected as the representative expression level for the gene. To account for potential batch effects across different experiments, we implemented the ComBat function from the R package sva [17]. We performed combined processing and analysis on the TCGA and GEO CRC cohorts.

### Differential gene expression

Differential expression analysis, employing the limma package, allowed us to discern the genes with differential expression (DEGs) between tumor and normal tissues. Heatmap and volcano plot visualizations of the DEG results were created using the heatmap and ggplot2 packages [18], respectively. Conducting univariate COX regression analysis allowed us to pinpoint DEGs that are associated with patient prognosis. Furthermore, the igraph package was utilized to construct a correlation network map of prognostic-related DEGs [19].

### Copy number alteration analysis

We defined copy number variations (CNVs) with values greater than 0 as CNV amplifications and those less than 0 as CNV deletions. We then calculated the percentage of CNV amplifications and deletions among the DEGs. To further investigate the genomic locations of CNV alterations in adipogenesis-related DEGs, we plotted the positions of these CNV changes across the 23 chromosomes.

### Consensus clustering analysis

Using the R package ‘ConsensusClusterPlus‘ [20], we characterized different molecular subtypes based on ARG expression. To ensure the robustness of the classification, we repeated the cluster determination process 1000 times in the colorectal cancer cohort. Visualizing the distribution differences of ARGs subtypes was achieved through Principal Component Analysis (PCA). To evaluate the clinical importance of these subtypes, we explored their associations with clinical factors, including prognosis, age, gender, and stage. Comparisons of overall survival (OS) among different clusters were performed using Kaplan-Meier survival curves, with a significance level set at P ≤ 0.05. Additionally, we employed the heatmap R package [21] to generate a heatmap representing the expression patterns of ARGs, which exhibited significant differences among the subgroups (P ≤ 0.05).

### Construction of risk score

The glmnet package [20] was utilized for Lasso Cox regression analysis, integrating data from both TCGA and GEO cohorts, to identify the most promising prognostic biomarkers. We utilized ten-fold cross-validation to construct models and confidence intervals for each lambda and eventually incorporated the most optimal key prognostic genes to build the risk model. To create training and testing sets, we randomly divided the TCGA combined with the GEO cohort at a 1:1 ratio. Employing the survival R package [22], we computed risk scores for each patient and classified them into high and low-risk groups using the median value. We used the ‘pROC’ package [23] to generate receiver operating characteristic (ROC) curves and evaluate the accuracy of the risk model. Furthermore, Kaplan-Meier survival analysis was executed on patients in the high and low-risk groups and 1-year, 3-year, and 5-year ROC curves were plotted using the ‘timeROC’ package [24], incorporating data from the training and testing sets. Through univariate and multivariate Cox regression analyses, we assessed the capacity of the risk model to function as an independent prognostic factor.

### GSEA and GSVA analysis

The R programming language was utilized for Gene Set Enrichment Analysis (GSEA) by employing the “enrichplot“ [25] and “clusterProfiler” packages [26]. Two predefined gene sets, namely “c5.go.symbols.gmt” and “c2.cp.keg.symbols.gmt,“ were employed for the analysis. The primary objective of the enrichment analysis was to identify significantly enriched pathways based on a P-value threshold of < 0.05. To elucidate the biological relevance of the gene sets in the context of various biological processes and disease progression, we employed the ‘GSVA’ package [27] to conduct Gene Set Variation Analysis (GSVA). Significant differences in gene set variation were determined using a significance threshold of |t| > 2 and a P-value < 0.05.

### Establishment of a nomogram

A column line plot was generated using the outcomes from multivariate Cox regression analysis to enable visual risk prediction. Each factor was assigned a score, and the column line plot depicted the cumulative risk score for each individual. This plot allows for a visual assessment of the predicted risk levels across the study cohort. Both the nomogram and calibration plot were generated using the rms package in R software [28].

### Immune-related features and prediction of immunotherapeutic

Quantification of immune cell infiltration levels in each cancer sample was accomplished using expression data, facilitating a comparison of cell infiltration level differences across various sample classifications. Additionally, we used the “e1071“ [29], “CIBERSORT“ [30], and “parallel” packages [31] in R software to calculate the immune cell infiltration patterns in each tumor sample. Following that, we employed the ‘Limma’ R software to examine the variations in immune cell enrichment between the high and low-risk groups. Additionally, we integrated a series of predictive indicators for immune checkpoint inhibitor (ICI) response, including immune checkpoints, tumor immune dysfunction, and exclusion (TIDE) scores, to examine the connection between the risk model and the efficacy of immune therapy.

### In vitro cell culture

We sourced colorectal cancer cell lines (SW480 and HCT116) and normal intestinal epithelial cell line (NCM460) from the Cell Repository of the Chinese Academy of Sciences (Shanghai, China). For all cell types, culture conditions involved the use of Dulbecco’s Modified Eagle Medium (DMEM) supplemented with 10% fetal bovine serum (FBS) and 1% penicillin-streptomycin solution, and maintenance in a 5% CO2 atmosphere at 37 °C.

### qRT-PCR

RNA extraction from the cell lines was performed using TRIzol reagent (Invitrogen), followed by cDNA synthesis using the PrimeScript RT Reagent Kit (Takara, China) with the obtained RNA. Subsequently, qPCR was conducted on the Step One Plus Real-Time PCR system (Applied Biosystems, Carlsbad, CA, USA) under the following conditions: an initial denaturation step at 95 °C for 10 min, followed by 40 cycles of denaturation at 95 °C for 15 s and annealing/extension at 60 °C for 1 min. The list of gene primers was found in supplementary file.

### Drug sensitivity analysis

In order to identify potential tumor treatment targets and improve tumor treatment, we utilized the Tumor Drug Sensitivity Multiomics Database (GDSC Database https://www.cancerrxgene.org/) We downloaded the IC50 values of 198 drugs and used the “oncoPredict” package to predict the IC50 of each sample in TCGA based on mRNA gene expression data. Applying Spearman correlation analysis to search for potential effective drugs for the treatment of colorectal cancer.

### Statistical analysis

Using the ‘gsurvplot’ function, we generated Kaplan-Meier survival curves and evaluated the differences in overall survival time between diverse groups via two-sided log-rank tests. By employing the risk-scoring formula, we divided cancer patients into high-risk or low-risk subtypes, with the median as the cutoff. To identify characteristics significantly associated with overall survival, we performed univariate Cox regression analysis. Subsequently, multivariate Cox regression analysis was conducted to assess whether these characteristics remained independent of other clinical features. All statistical analyses were performed using R software (version 4.1.3), and p-values < 0.05 were considered statistically significant.

## Results

### Overview of the expression and genetic variation of ARGs in CRC

To investigate the impact of ARGs on the occurrence and progression of CRC, we analyzed the RNAseq data of normal and tumor tissues from the TCGA database. We identified DEGs using a threshold of FDR < 0.05 and an absolute logFC greater than 1. This analysis led to the identification of 50 differentially expressed ARGs, which were visualized through a heatmap and a volcano plot (Figure S2A, B). We further performed univariate Cox analysis on the DEGs to determine their association with overall survival in CRC patients. The results revealed that a total of 13 differentially expressed ARGs were significantly associated with patient survival outcomes, with 8 ARGs associated with unfavorable survival outcomes (Figure S2C). The prognostic relevance and interrelationships of these ARGs were summarized in a prognostic-related network depicted in Figure S2D. Additionally, we analyzed copy number variations (CNVs) to further explore ARG mutations in colorectal cancer. We determined the occurrence rate of ARG mutations by integrating CNV data and identified 45 ARGs with CNV alterations (Figure S2E), mostly characterized by copy number amplifications. The genomic locations of these CNV alterations are shown in Figure S2F. These results shed light on the potential prognostic value of ARGs in colorectal cancer and demonstrate their association with patient survival outcomes. The analysis of CNV alterations provides additional insights into the occurrence and distribution of mutations in ARGs, which may contribute to a deeper understanding of the molecular mechanisms underlying CRC development.

### Identification of ARGs subtypes

**Fig. 1:**
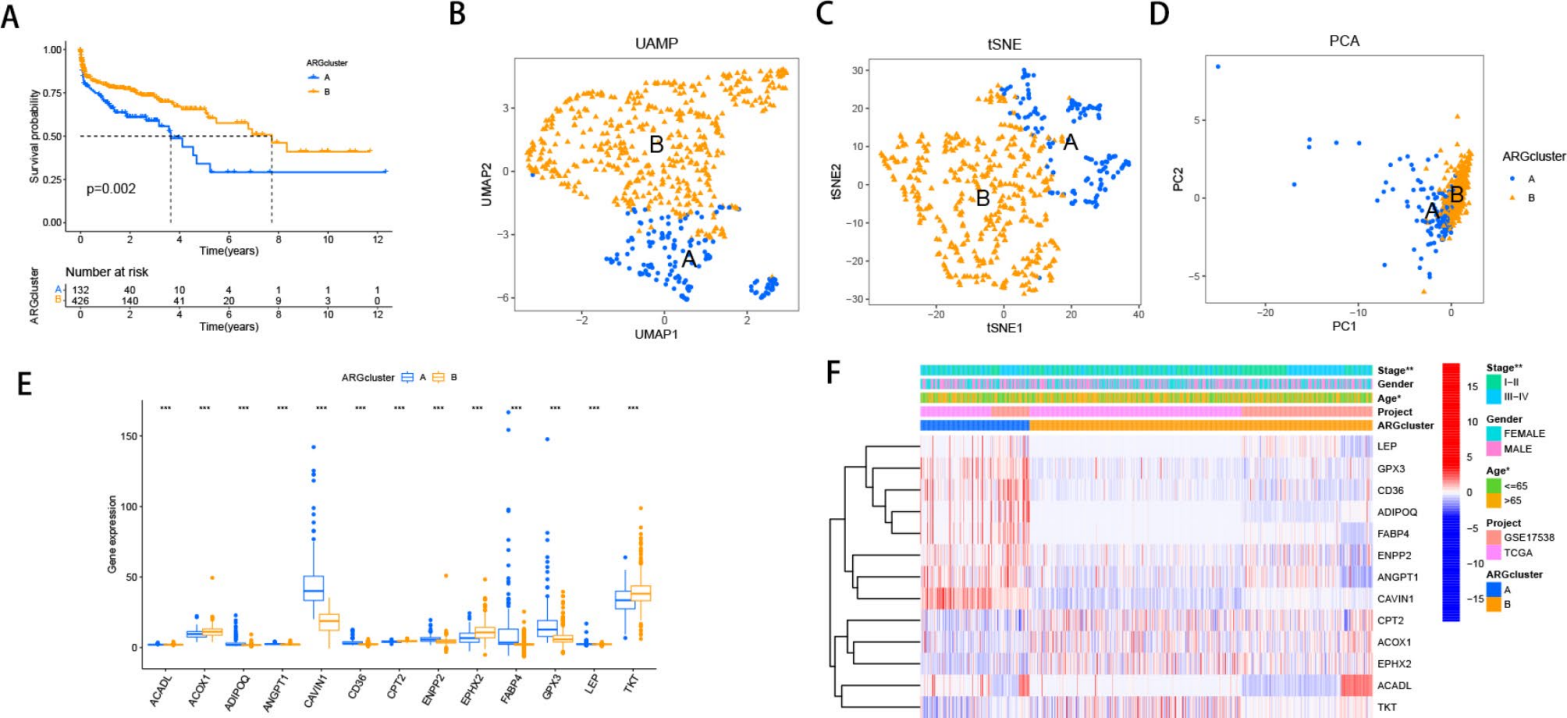
Molecular subtypes based on ARGs in CRC and their clinicopathological features. (**A**) Kaplan-Meier survival analyses for the two molecular subtypes. UAMP(**B**), tSEN(**C**), and PCA(**D**) presented a great difference between the A and B subtypes. (**E**)The expression levels of prognosis related differentially expressed ARGs between A and B subtypes. (**F**) The heatmap showed the prognosis related differentially expressed ARGs expression profiles and clinicopathologic characteristics among subtypes A and B

We employed unsupervised cluster analysis to stratify CRC patients’ samples based on ARGs expression levels, leading to the identification of two distinct clusters, termed Cluster A and Cluster B (Figure S3). Survival analysis indicated a favorable survival advantage for patients in Cluster B (Fig. 1A). Subsequently, we applied three common dimensionality reduction techniques, namely PCA, t-SNE, and UMAP, to visualize the data, with each cell population represented by different colors. The visualizations clearly distinguished Cluster A and Cluster B (Fig. 1B-D). Differential expression analysis identified 13 ARGs with significant expression differences between the two clusters, including 9 upregulated and 4 downregulated in Cluster A compared to Cluster B (Fig. 1E). Notably, the clinical characteristics also exhibited significant differences between the two subtypes, with patients in Cluster A displaying older age and higher STAGE staging (Fig. 1F).

**Fig. 2:**
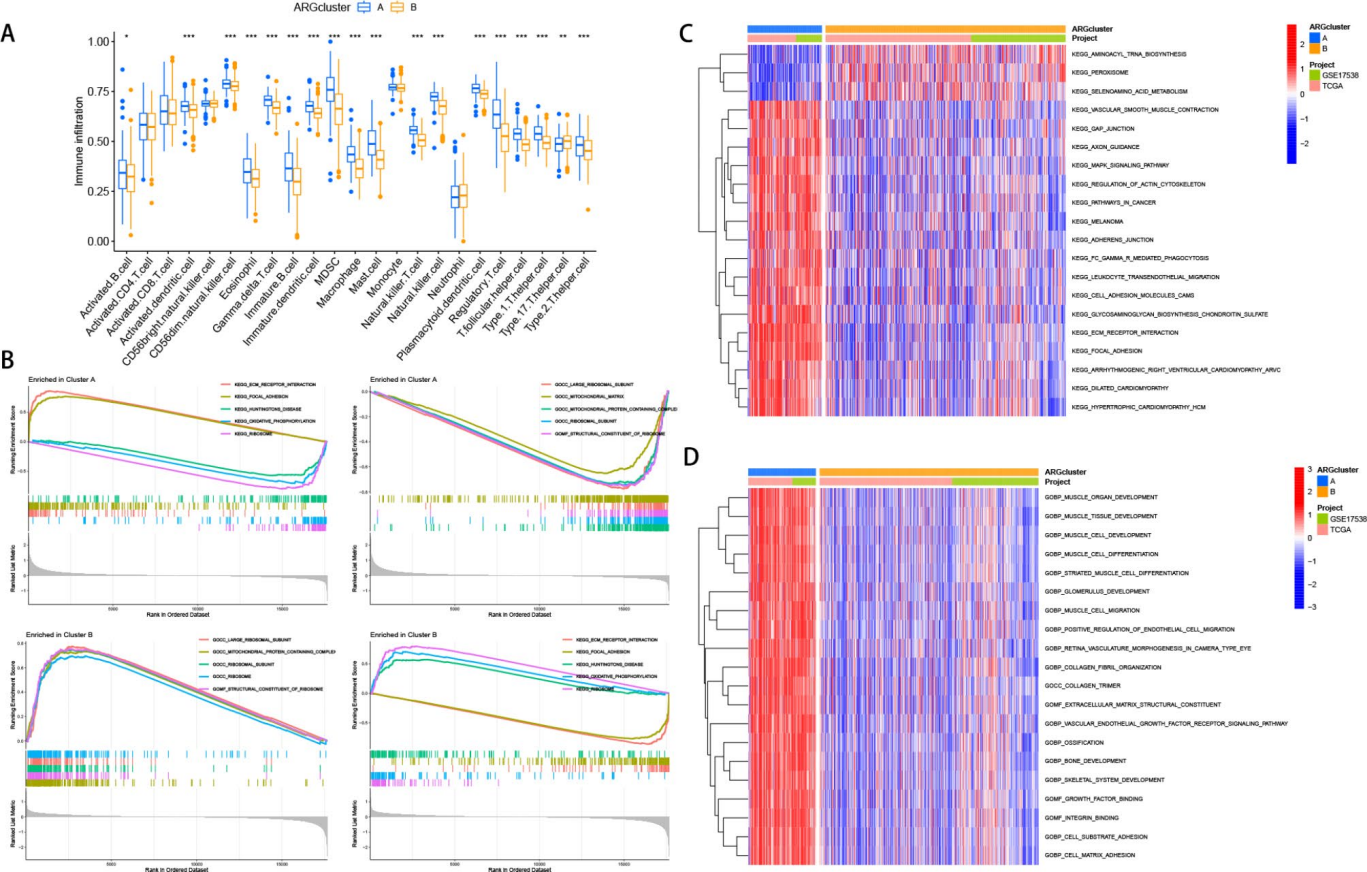
Tumor microenvironment and Functional enrichment analysis of ARGs-based clusters in CRC. (**A**) Analysis of infiltrating immune cells between A and B subtypes. (**B**) Analysis of GSEA for subtypes A and B. **C, D.** Analysis of GSVA for subtypes A and B

To explore the immune microenvironment of Clusters A and B in CRC, we performed ssGSEA to compare the expression profiles of 23 immune cell subtypes between the two clusters. The results revealed that 18 out of 23 immune cell subtypes exhibited differential expression between the clusters (Fig. 2A). Furthermore, to gain insights into the underlying biological alterations associated with the distinct clusters, we conducted GSEA and GSVA analyses. GSEA unveiled significant activation of ECM-receptor interaction and Focal adhesion pathways in Cluster A, whereas ribosome and oxidative phosphorylation-related metabolic pathways were enriched in Cluster B (Fig. 2B). GSVA analysis of the two subtypes indicated that highly expressed DEGs in Cluster A were significantly enriched in cell communication and information pathways, MAPK signaling pathway, and cell adhesion-related pathways (Fig. 2C, D).

### Metabolic reprogramming induced by ARGs subtypes

The metabolic pathway covers various important biological molecule synthesis and decomposition processes within cells, which are crucial for normal cell function and survival. At the same time, dysmetabolism is also a core feature of cancer. The further screening of metabolism-related pathways through GSEA reveals that the molecular subtypes of CRC based on ARGs also reflect the metabolic alterations involved in the progression of colorectal cancer. As shown in Additional Table 1, and 2, Besides fatty acid metabolism, Cluster B exhibits significantly enriched metabolic pathways, encompassing the following categories: Carbohydrate Metabolism (Butanoate Metabolism, Propanoate Metabolism, Pyruvate Metabolism, CITRATE CYCLE TCA CYCLE, Starch and Sucrose Metabolism), Nucleotide Metabolism (Pyrimidine Metabolism and Purine Metabolism), Amino Acid Metabolism (Arginine and Proline Metabolism, Cysteine and Methionine Metabolism), Drug Metabolism (Drug Metabolism-Other Enzymes and Drug Metabolism-Cytochrome P450), Vitamin Metabolism (Retinol Metabolism), Organic Acid Metabolism (Glyoxylate and Dicarboxylate Metabolism), and Other Metabolic Pathways (Porphyrin and Chlorophyll Metabolism, Glutathione Metabolism).

### Construction and validation of risk prognostic models

**Fig. 3:**
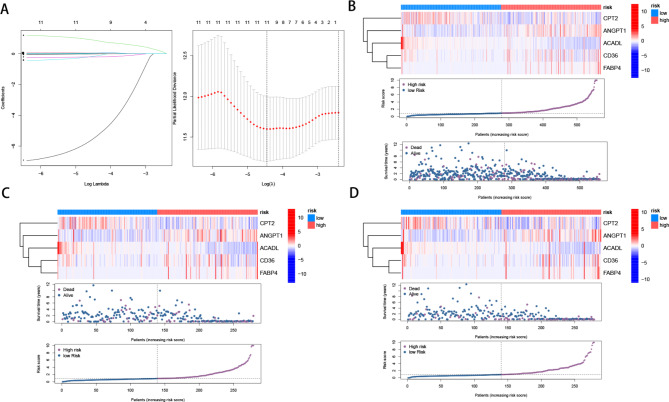
Construction of prognostic model based on ARGs in CRC (**A**) LASSO Cox regression analysis. Distribution of the heatmap of ARGs (upper), survival time (middle), and risk score (below) in all cohorts (**B**), training cohort(**C**), and test cohort(**D**)

**Fig. 4:**
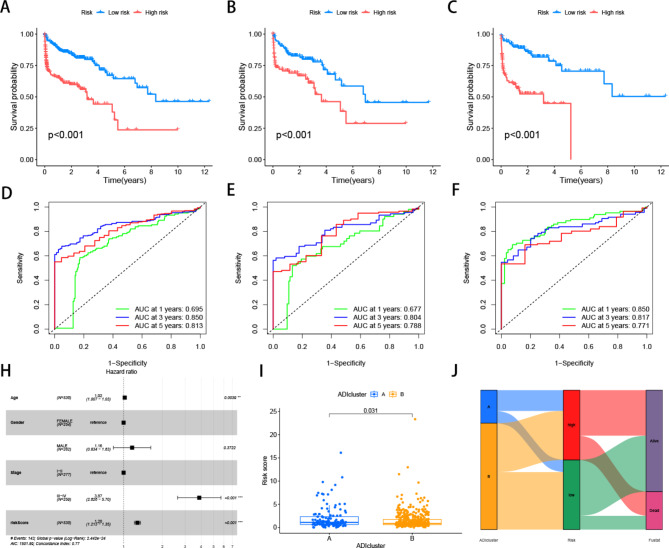
K-M survival curves of low- and high-risk patients in the training cohort(**A**), test cohort(**B**), and all cohort(**C**). **D-F.** The ROC curves at 1-, 3- and 5-year in the mentioned three cohorts. **H.** Forest plots showing the results of the multivariate Cox regression analysis. **I.** Differences in risk scores between clusters A and B **J.** Sankey diagram shows the relationship between different ARG scores, risk scores, and survival outcomes

**Fig. 5:**
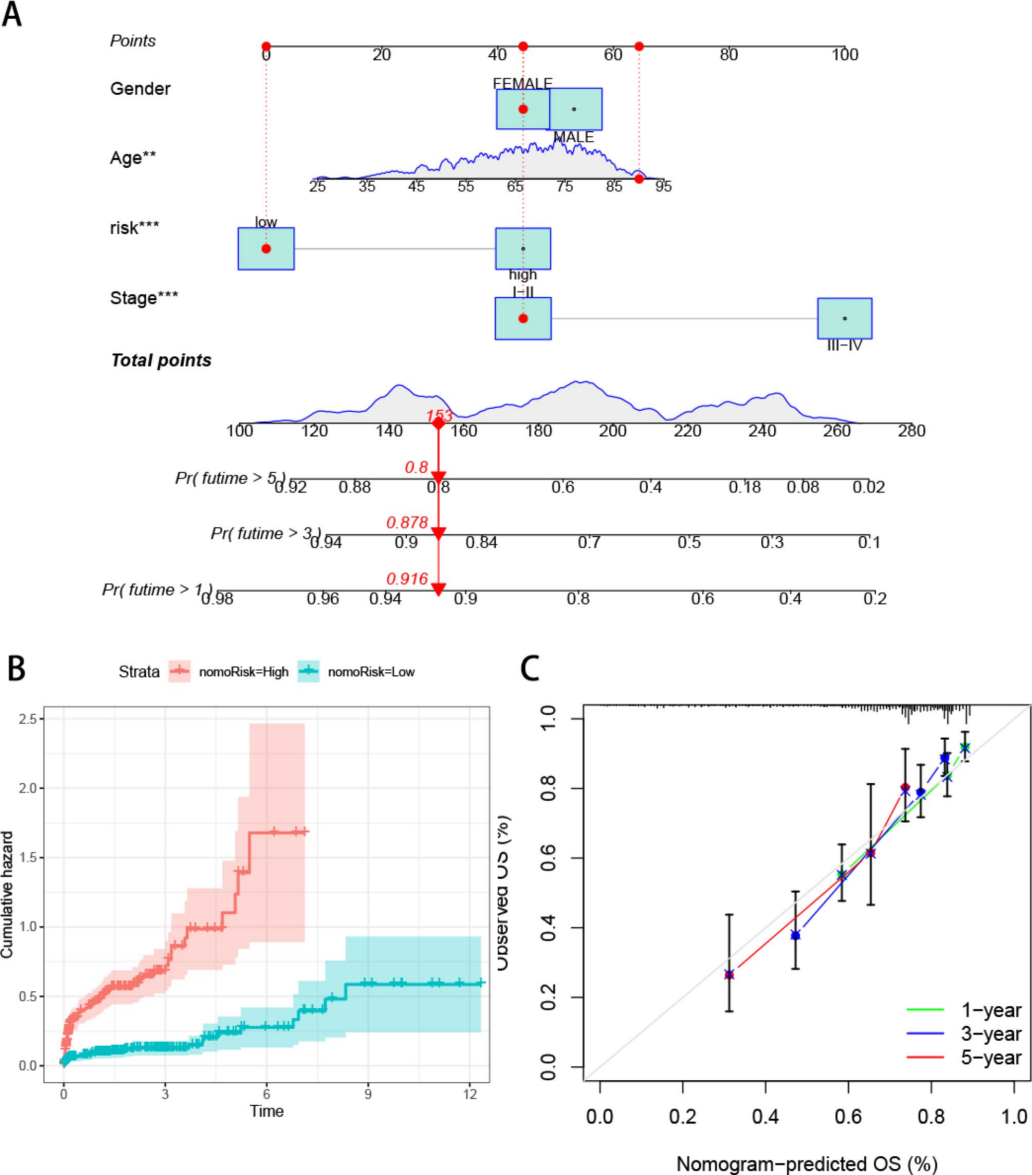
Construction and validation of a nomogram (**A**) Nomogram for predicting the 1-, 3-, and 5-year OS of CRC patients. (**B**) Draw a cumulative risk map for high and low-risk groups (**C**) Calibration plots show the fits of 1-, 3- and 5-year predictions

To establish a feature-scoring model for evaluating the role of ARGs in CRC, we employed LASSO regression analysis to select the best prognostic feature-related genes from prognostic-related key genes. After incorporating the variables into the LASSO regression model with minimized λ, five ARGs were selected to construct the risk-scoring model (Fig. 3A). Initially, the samples were stratified into train and test groups with a 1:1 ratio. Figure 3B-D depicted the gene expression levels and survival time, along with the status distribution between the high and low-risk groups in various groups. The results showed an increasing proportion of patient mortality with elevated risk scores. Simultaneously, survival analysis across the three groups revealed that patients with higher risk scores exhibited significantly inferior overall survival (OS) compared to those with lower risk scores (Fig. 4A-C). Time-dependent ROC analysis for 1-year (train group 0.695, test group 0.677, all samples group 0.850), 3-year (train group 0.850, test group 0.814, all samples group 0.817), and 5-year (train group 0.813, test group 0.788, all samples group 0.771) overall survival further validated the robust predictive capacity of the ARGs-associated risk model for colon cancer patient survival (Fig. 4D-F). Subsequent multivariable Cox regression results indicated that the risk score independently served as a prognostic factor for OS (Fig. 4H). Patients in the A and B clusters also demonstrated differences in risk scores, with Cluster A patients exhibiting higher risk scores, corresponding to a poorer prognosis for previous patients in Cluster A (Fig. 4I). Sankey plots illustrating both groups displayed the correlation between ARGs risk model grouping, ARGs subtypes, and survival status. The high-risk group displayed a higher proportion of fatal outcomes compared to the proportion of alive patients, and within Cluster A, the proportion of high-risk patients was higher than that of low-risk patients (Fig. 4J). Based on the results of univariate and multivariate Cox regression analyses, we constructed a nomogram incorporating clinical staging and ARGs (Fig. 5A). The cumulative graph demonstrated a significant distinction between high and low-risk groups over time (Fig. 5B). Calibration curves indicated the high accuracy of the nomogram (Fig. 5C). These findings collectively suggest that ARGs possess a reliable capacity to discriminate tumor outcome differences.

### Tumor mutation burden and immune landscape of ARGs subtypes

The current investigation employed the Maftools software package to undertake a comparative analysis of somatic mutations between high and low-risk oncology cohorts. Our analysis revealed that the high-risk cohort (Figure S4A) displayed a significantly higher tumor somatic mutation rate when compared to the low-risk cohort (Figure S4B). Further identification of tumor driver genes in both cohorts indicated a higher number of such genes in the low-risk group (Figure S4C, D). The analysis of co-occurring and mutually exclusive mutations in the high and low-risk cohorts showcased the correlation among mutated genes, with green and orange colors representing co-occurrence and mutual exclusivity, respectively (Figure S4E, F). Moreover, the forest plot illustrating differentially mutated genes between the high and low-risk cohorts (Figure S5) indicated a higher proportion of mutated genes in the low-risk group.

### Immunogenomic patterns and immunotherapy analysis

**Fig. 6:**
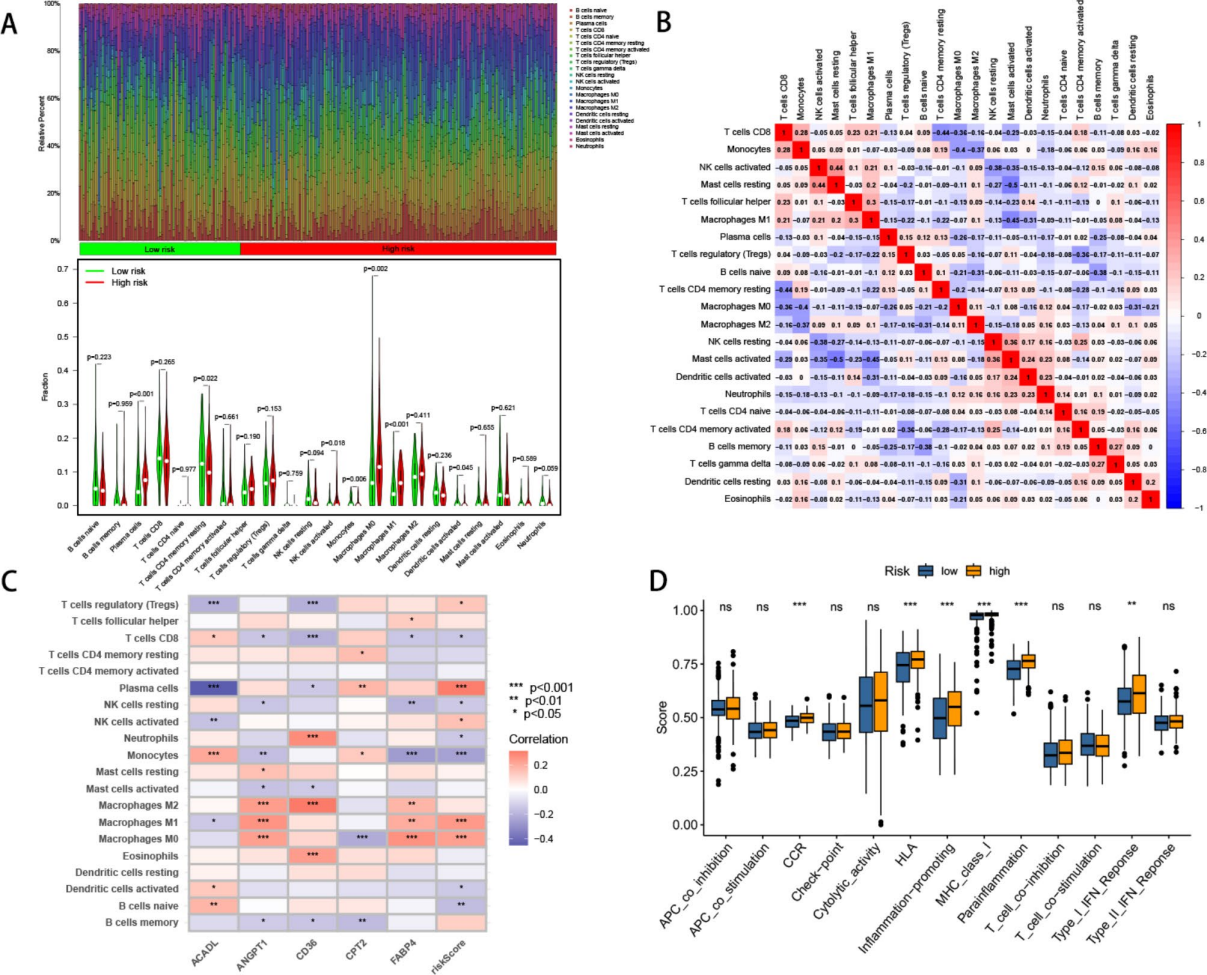
Tumor microenvironment features related to the ARGs-based signature in CRC. (**A**) The proportions of immune cells in each sample of low- and high-risk groups according to CIBERSORT analysis. (**B**) The correlation of immune cells (**C**) The heatmap showed the relationship between ARGs and immune cells. (**D**) Immune function analysis

Using the CIBERSORT method, we determined the proportions of 22 immune infiltrating cell types in each sample and subsequently investigated the differential expression of immune cells between the high and low-risk groups. Our findings demonstrated that the high-risk group exhibited a higher proportion of resting immune cells (Fig. 6A). Furthermore, the correlation analysis of the 22 immune cell types revealed positive correlations indicated in red and negative correlations indicated in blue (Fig. 6B). Leveraging these results, we examined the relationship between genes involved in model construction and immune cells, observing that most genes showed negative correlations with immune cells (Fig. 6C). Interestingly, we observed a higher immune functionality in the high-risk group (Fig. 6D).

**Fig. 7:**
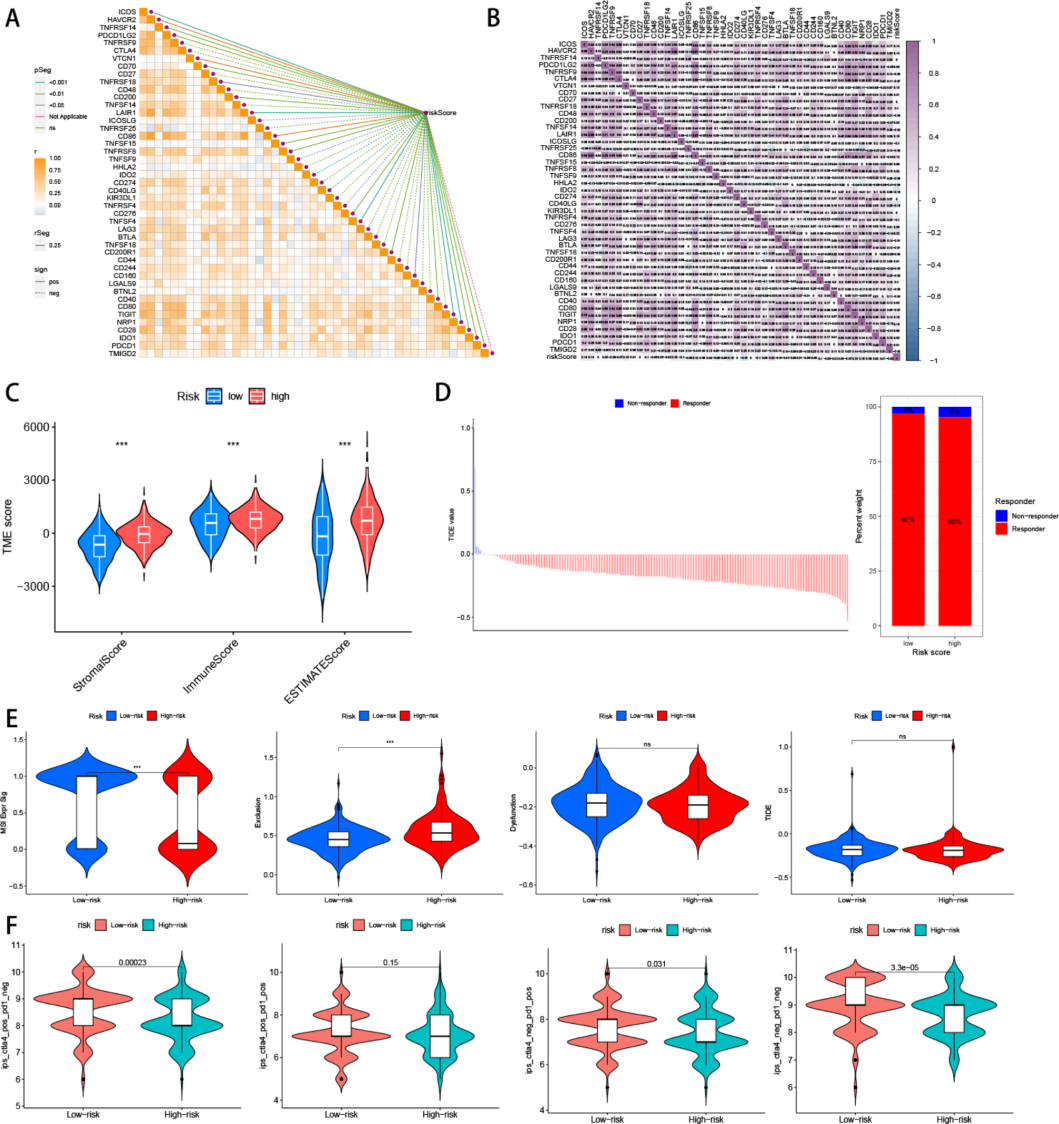
Immune checkpoint profiles and immunotherapy evaluation related to the ARGs-based signature in CRC. **A, B.** correlations between the immune checkpoint expression and risk score. **C.** Comparison of TEM scores between the high and low-risk groups. **D.** analyze immunotherapy responses in high and low-risk groups by TIDE **E.** Predictive immunotherapy by comparing the score of MSI, Exclusion, Dysfunction, and TIDE in high and low-risk groups. **F.** Immunotherapy response analysis of high and low-risk groups through TCIA

To evaluate the responsiveness of patients in the high and low-risk groups to immunotherapy, we initially conducted a risk score analysis and explored the correlation of immune checkpoint molecules. Remarkably, we identified ten immune checkpoint molecules, including ICOS, HAVCR2, CTCN1, etc. which displayed significant positive correlations, while five immune checkpoint molecules, including ICOSLG, TNFSF9, IDO2, CD40LG, and TNFRSF4, showed significant negative correlations (Fig. 7A, B). Additionally, ESTIMATE analysis revealed that the high-risk group exhibited higher immune, stromal, and ESTIMATE scores compared to the low-risk group (Fig. 7C). Subsequently, we employed the Tumor Immune Dysfunction and Exclusion (TIDE) algorithm (http://tide.dfci.harvard.edu/) to assess the efficacy of immunotherapy in high and low-risk populations. The results depicted in Fig. 7D indicated a higher proportion of immunotherapy-resistant patients in the high-risk group. Furthermore, the high-risk group displayed lower microsatellite instability (MSI) scores, higher TIDE scores, and T cell exclusion scores, with no significant difference in T cell dysfunction compared to the low-risk group (Fig. 7E). Finally, through the evaluation of PD1 and CTLA-4, we found that patients in the low-risk group may exhibit a more favorable response to immunotherapy (Fig. 7F).

### Drug response analysis

In order to identify potentially effective drugs for treating colorectal cancer, we examined the correlation between drug sensitivity (IC50 values) and gene expression profiles in high-risk and low-risk groups. Our analysis revealed that several drugs exhibited promising effects on colorectal cancer patients. Specifically, 39 drugs were identified as having potential efficacy in the treatment of colorectal cancer. Some of these drugs include Afuresertib, AGI-5198, Crizotinib, Dabrafenib, Dasatinib, and many others. Our analysis revealed that certain drugs, such as Dasatinib, Doramapimod, JQ1, and NU7441, demonstrated greater efficacy in the low-risk subgroup of patients (Figure S6). On the other hand, the remaining drugs showed higher efficacy in the high-risk subgroup of patients. These findings suggest that different patient risk profiles may influence the response to specific drugs, highlighting the importance of personalized medicine approaches in tailoring treatment strategies for colorectal cancer patients. Further investigation is warranted to understand the underlying mechanisms and potential biomarkers associated with drug response in different risk groups.

### Validation of key genes expression in tumor cell lines in vitro

We detected the expression of 5 ARGs (Cd36, FABP4, ANGPT1, ACADL, and CPT2) in CRC cell lines (SW480 and HCT116) and normal intestinal epithelial cells (NCM460) using RT-qPCR in vitro. Except for ANGPT1, the expression trends of the other ARGs were consistent with those in TCGA (Figure S7).

## Discussion

We acknowledge the potential association between fat synthesis and CRC, our research is warranted to gain a comprehensive understanding of the specific roles and interactions of these genes.

We comprehensively analyzed 200 ARGs in CRC by employing RNAseq data from both normal and tumor tissues acquired from the TCGA and GEO databases. Through differential expression analysis, we identified 50 differentially expressed ARGs associated with CRC. A total of 40 ARGs were identified to be upregulated. The results of further univariate Cox analysis unveiled 13 differentially expressed ARGs significantly linked to patients’ survival outcomes in CRC. Moreover, we demonstrated that there is a complex correlation between 13 differentially expressed ARGs related to prognosis. These findings emphasize the potential importance of ARGs in determining the prognosis of CRC patients.

How are ARGs des-regulated in CRC? Research indicates that genetic variations associated with fat metabolism may influence an individual’s degree of obesity and the expression of genes. We explored the genetic variations of ARGs in CRC by analyzing CNVs, this analysis identified a high mutation percentage of ARGs (45/50) with CNV alterations and mostly characterized by copy number amplifications. The presence of these CNV alterations in ARGs may contribute to the dysregulation of adipogenesis-related pathways, thereby influencing colorectal cancer development.

Furthermore, unsupervised cluster analysis based on ARGs expression levels led to the identification of two distinct subtypes, Cluster A and Cluster B. Patients in Cluster B exhibited a favorable survival advantage compared to those in Cluster A, suggesting that these ARGs may play a role in defining the molecular characteristics of the subtypes in CRC.

What are the reasons for the survival differences between two different subtypes based on ARGs in colorectal cancer patients? In subtype A with poorer prognosis, we found most ARGs(9/13) are upregulated in tumor tissue. Abnormal expression of these ARGs may affect tumor development through multiple pathways (immune, metabolic, and other signaling pathways).

The process of fat synthesis is a biochemical process in which the body converts glucose and other metabolites into fatty acids and stores them in fat cells. This process is necessary under normal circumstances, but excessive fat synthesis can lead to various problems such as non-alcoholic fatty liver disease (NAFLD) and insulin resistance. Insulin resistance reduces the response of the body’s cells to insulin, which can stimulate the liver to synthesize more fatty acids and result in the accumulation of fat in the liver, exacerbating the progression of NAFLD. Research suggests that individuals with pre-existing NAFLD may have a higher incidence of CRC and liver metastasis [32]. In our study, colorectal cancer subtypes based on genes related to fat synthesis exhibited disruptions in multiple metabolic pathways, including sugar and lipid metabolism, nucleotide metabolism, amino acid metabolism, and drug metabolism pathways, among others. Understanding the relationship between genes associated with fat synthesis and their involvement in metabolic pathways in the development of tumors can aid in the development of new therapeutic approaches aimed at disrupting the energy supply and growth of tumors.

Glycolipid metabolism plays a crucial role in regulating immune function, as the activities within the immune system require energy support. Under normal circumstances, Glycolipid metabolism provides the necessary energy for immune cells, ensuring their effective execution of tasks. Moreover, sugar and lipid metabolism not only influence the energy supply of immune cells but also impact their differentiation and functionality. Further analysis suggests that there is a higher proportion of immune cells and more activation of tumor-related signaling pathways in subtype A compared with subtype B. The immune microenvironment alteration and various biological pathways activation may contribute to the different outcomes observed in the two subtypes.

Can ARGs stratify CRC patients’ risk and predict their prognosis? We conducted LASSO regression analysis and selected five hub ARGs to construct a risk-scoring model. The model successfully stratified patients into high and low-risk groups, with patients in the high-risk group showing significantly inferior overall survival. The predictive capacity of the model was further validated using time-dependent ROC analysis, demonstrating its robustness in predicting patient outcomes. Notably, the risk score independently served as a prognostic factor for overall survival, highlighting the potential clinical relevance of ARGs in predicting colorectal cancer prognosis.

Further research has found that patients in different risk groups have different immune microenvironments and reactions to immunotherapy, which will provide theoretical guidance and ideas for the development of immunotherapy based on patients in different risk groups.

Furthermore, the drug response analysis also identified potentially effective drugs for treating colorectal cancer. The analysis indicated that specific drugs showed greater efficacy in the low-risk subgroup of patients, while others showed higher efficacy in the high-risk subgroup. These findings suggest that considering patient risk profiles and ARG expression levels may be essential in tailoring personalized treatment strategies for colorectal cancer patients.

To validate and fully comprehend the role of ARGs in colorectal cancer, as well as to explore their therapeutic potential in clinical settings, further experimental validation and functional studies are essential. These efforts will be crucial in advancing our understanding and facilitating the translation of our findings into effective clinical interventions.

Our findings provide insights into the molecular mechanisms driving colorectal cancer development. This knowledge has the potential to guide the development of targeted therapies tailored to individual patients based on their ARGs expression profiles and risk scores.

### Electronic supplementary material

Below is the link to the electronic supplementary material.

Supplementary Material 1

Supplementary Material 2

## Data Availability

The data used and analyzed in this article are available from https://portal.gdc.cancer.gov/ and https://www.ncbi.nlm.nih.gov/geo/.
