# Supplementary material for: Comprehensive characterization of adipogenesis-related genes in colorectal cancer for clinical significance and immunogenomic landscape analyses
Source: Lipids Health Dis. 2023 Dec 7;22:217. doi: 10.1186/s12944-023-01942-9 (PMC10702012; doi:10.1186/s12944-023-01942-9)
Supplement: Supplementary file 1 — Supplementary Material 1 [file 12944_2023_1942_MOESM1_ESM.docx]

TABLE 1. The metabolic reprogramming caused by cluster B

| Description | setSize | enrichmentScore | NES | pvalue | p.adjust | qvalue |
| --- | --- | --- | --- | --- | --- | --- |
| KEGG_BUTANOATE_METABOLISM | 33 | 0.722712 | 2.079507 | 1.33E-05 | 9.56E-05 | 4.33E-05 |
| KEGG_CITRATE_CYCLE_TCA_CYCLE | 30 | 0.734212 | 2.083121 | 2.52E-05 | 0.000166 | 7.54E-05 |
| KEGG_PYRIMIDINE_METABOLISM | 91 | 0.512476 | 1.797634 | 7.43E-05 | 0.000399 | 0.000181 |
| KEGG_PROPANOATE_METABOLISM | 31 | 0.69665 | 2.007879 | 0.000138 | 0.000708 | 0.00032 |
| KEGG_PURINE_METABOLISM | 145 | 0.44703 | 1.654078 | 0.00014 | 0.000708 | 0.00032 |
| KEGG_RETINOL_METABOLISM | 45 | 0.594854 | 1.830117 | 0.00024 | 0.001087 | 0.000492 |
| KEGG_GLYOXYLATE_AND_DICARBOXYLATE_METABOLISM | 16 | 0.759032 | 1.843276 | 0.001612 | 0.005333 | 0.002415 |
| KEGG_ARGININE_AND_PROLINE_METABOLISM | 48 | 0.544722 | 1.69477 | 0.002695 | 0.007857 | 0.003558 |
| KEGG_PYRUVATE_METABOLISM | 39 | 0.569275 | 1.703429 | 0.004142 | 0.011491 | 0.005204 |
| KEGG_FATTY_ACID_METABOLISM | 40 | 0.547302 | 1.653685 | 0.005995 | 0.015163 | 0.006867 |
| KEGG_CYSTEINE_AND_METHIONINE_METABOLISM | 34 | 0.558239 | 1.606829 | 0.006106 | 0.015222 | 0.006894 |
| KEGG_DRUG_METABOLISM_OTHER_ENZYMES | 37 | 0.549947 | 1.630562 | 0.007645 | 0.01777 | 0.008047 |
| KEGG_STARCH_AND_SUCROSE_METABOLISM | 35 | 0.518597 | 1.507328 | 0.011171 | 0.024952 | 0.0113 |
| KEGG_DRUG_METABOLISM_CYTOCHROME_P450 | 57 | 0.462399 | 1.496919 | 0.014912 | 0.031664 | 0.01434 |
| KEGG_METABOLISM_OF_XENOBIOTICS_BY_CYTOCHROME_P450 | 54 | 0.474352 | 1.520042 | 0.015669 | 0.032591 | 0.01476 |
| KEGG_PORPHYRIN_AND_CHLOROPHYLL_METABOLISM | 28 | 0.522757 | 1.461694 | 0.032243 | 0.060281 | 0.0273 |
| KEGG_GLUTATHIONE_METABOLISM | 46 | 0.443265 | 1.376797 | 0.045092 | 0.079957 | 0.036211 |

TABLE 2. The metabolic reprogramming caused by cluster A

| Description | setSize | enrichmentScore | NES | pvalue | p.adjust | qvalue |
| --- | --- | --- | --- | --- | --- | --- |
| KEGG_BUTANOATE_METABOLISM | 33 | -0.72271 | -2.07687 | 6.57E-06 | 4.92E-05 | 2.29E-05 |
| KEGG_CITRATE_CYCLE_TCA_CYCLE | 30 | -0.73421 | -2.07331 | 3.29E-05 | 0.000202 | 9.40E-05 |
| KEGG_PURINE_METABOLISM | 145 | -0.44703 | -1.66132 | 7.37E-05 | 0.000409 | 0.00019 |
| KEGG_PROPANOATE_METABOLISM | 31 | -0.69665 | -1.98108 | 8.59E-05 | 0.000462 | 0.000215 |
| KEGG_PYRIMIDINE_METABOLISM | 91 | -0.51248 | -1.75109 | 0.000139 | 0.000683 | 0.000318 |
| KEGG_RETINOL_METABOLISM | 45 | -0.59485 | -1.80203 | 0.000482 | 0.001962 | 0.000913 |
| KEGG_GLYOXYLATE_AND_DICARBOXYLATE_METABOLISM | 16 | -0.75903 | -1.79263 | 0.001121 | 0.003707 | 0.001724 |
| KEGG_ARGININE_AND_PROLINE_METABOLISM | 48 | -0.54472 | -1.68456 | 0.001823 | 0.0055 | 0.002558 |
| KEGG_PYRUVATE_METABOLISM | 39 | -0.56927 | -1.66663 | 0.004169 | 0.011565 | 0.005379 |
| KEGG_FATTY_ACID_METABOLISM | 40 | -0.5473 | -1.61336 | 0.00563 | 0.014212 | 0.00661 |
| KEGG_CYSTEINE_AND_METHIONINE_METABOLISM | 34 | -0.55824 | -1.61942 | 0.007447 | 0.018041 | 0.008391 |
| KEGG_DRUG_METABOLISM_OTHER_ENZYMES | 37 | -0.54995 | -1.60183 | 0.010691 | 0.024196 | 0.011254 |
| KEGG_METABOLISM_OF_XENOBIOTICS_BY_CYTOCHROME_P450 | 54 | -0.47435 | -1.51848 | 0.013149 | 0.028628 | 0.013315 |
| KEGG_DRUG_METABOLISM_CYTOCHROME_P450 | 57 | -0.4624 | -1.49198 | 0.015981 | 0.033197 | 0.01544 |
| KEGG_STARCH_AND_SUCROSE_METABOLISM | 35 | -0.5186 | -1.50842 | 0.026835 | 0.051284 | 0.023853 |
| KEGG_PORPHYRIN_AND_CHLOROPHYLL_METABOLISM | 28 | -0.52276 | -1.45189 | 0.03922 | 0.072604 | 0.033769 |
| KEGG_GLUTATHIONE_METABOLISM | 46 | -0.44327 | -1.34933 | 0.039257 | 0.072604 | 0.033769 |
