# Supplementary material for: Comprehensive characterization of adipogenesis-related genes in colorectal cancer for clinical significance and immunogenomic landscape analyses
Source: Lipids Health Dis. 2023 Dec 7;22:217. doi: 10.1186/s12944-023-01942-9 (PMC10702012; doi:10.1186/s12944-023-01942-9)
Supplement: Supplementary file 2 — Supplementary Material 2 [file 12944_2023_1942_MOESM2_ESM.docx]

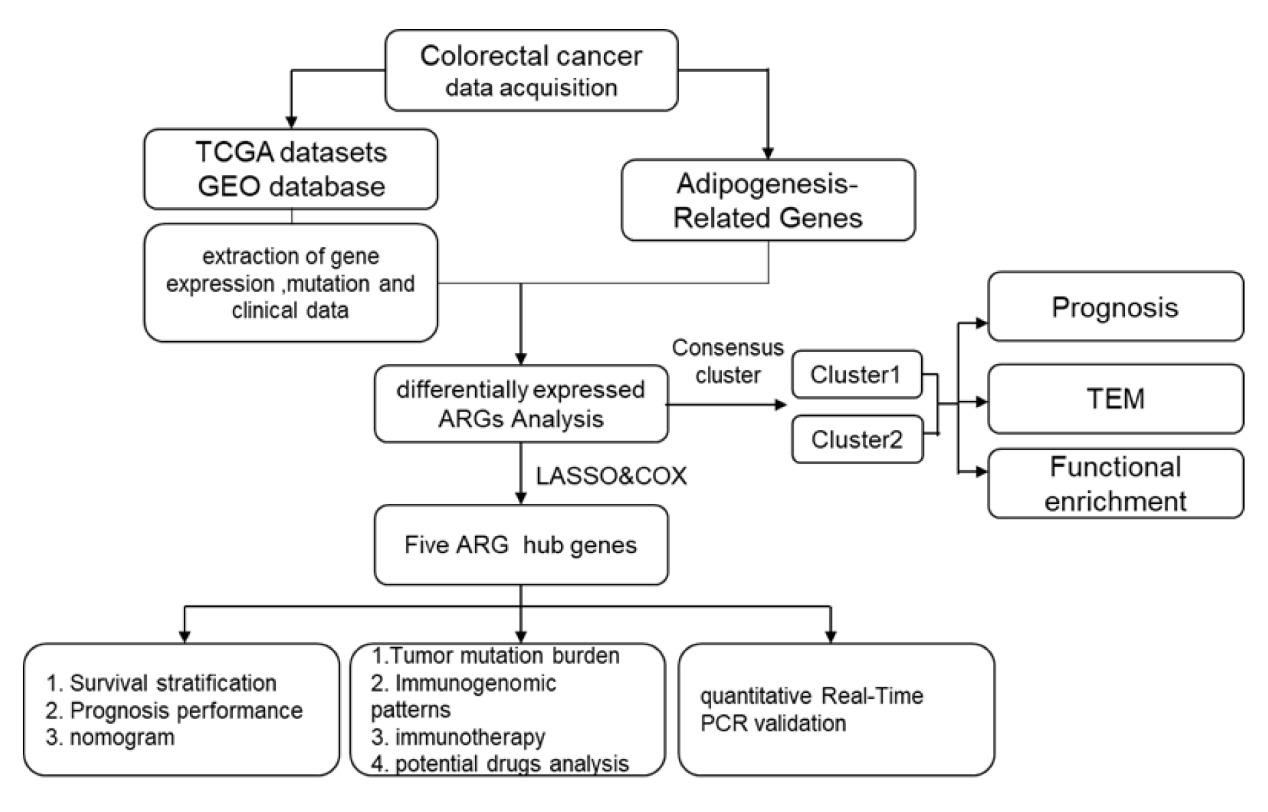


**Figure S1.** Study flow chart.


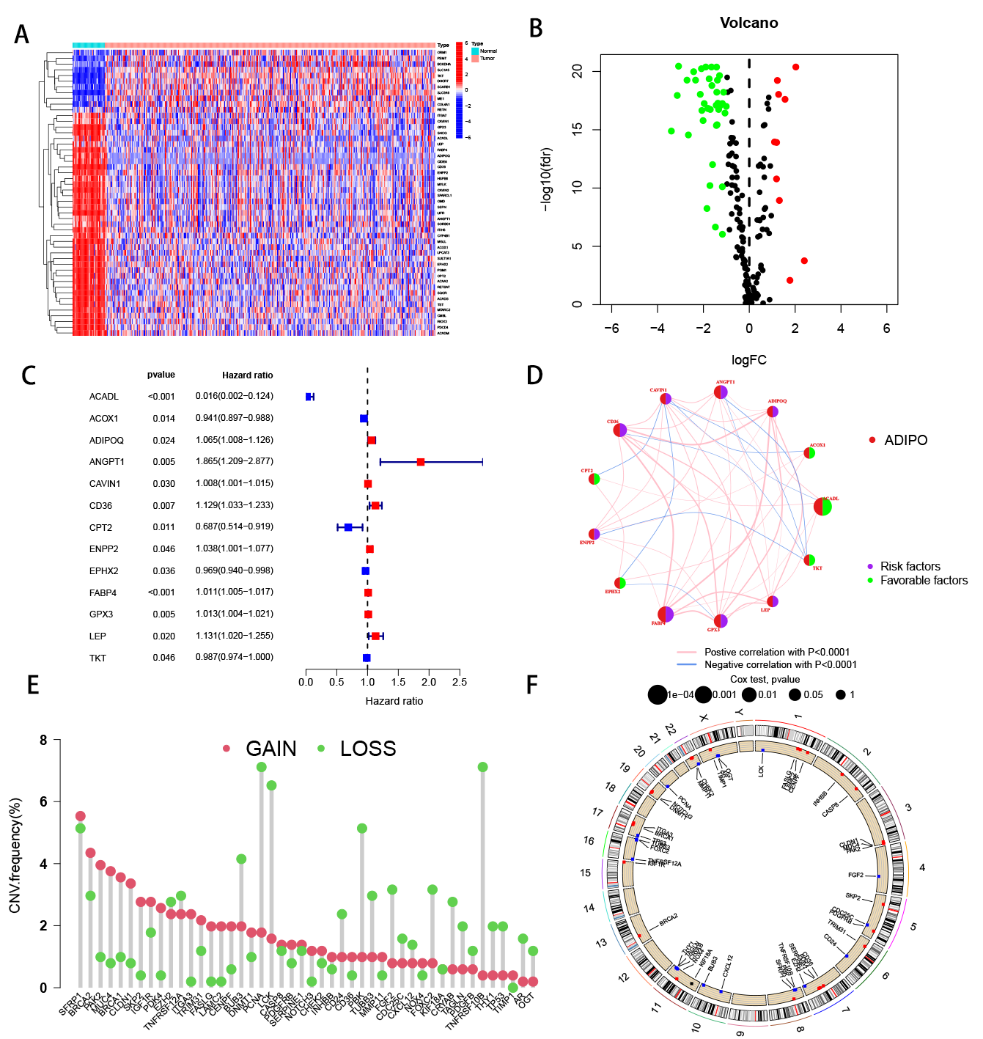


**Figure S2.** The transcriptional expression and genetic alterations of ARGs in CRC. **A.** Heatmap of the expression of top 50 DEGs in the cancer samples and normal samples. **B.** Volcano plots of DEGs between the above two groups. **C.** Univariate Cox regression analysis for screening the survival-related ARGs. **D.** The interaction and correlation of the prognosis related differentially expressed ARGs in CRC. **E.** The CNV frequency of ARGs **F.** Locations of CNV alterations on 23 chromosomes.


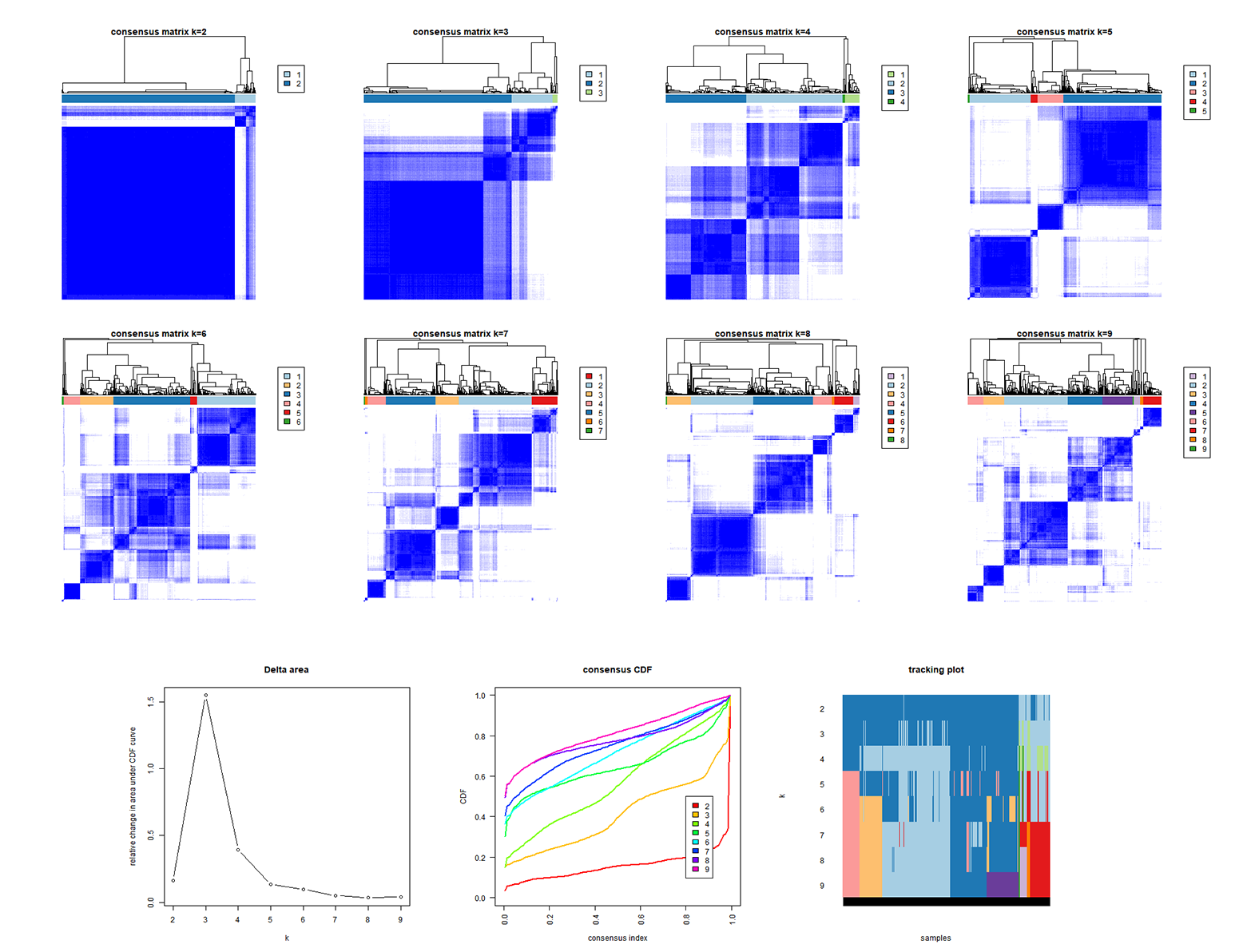


**Figure S3**. A. Identification of molecular subtypes based on the expression of ARGs through consensus clustering analysis


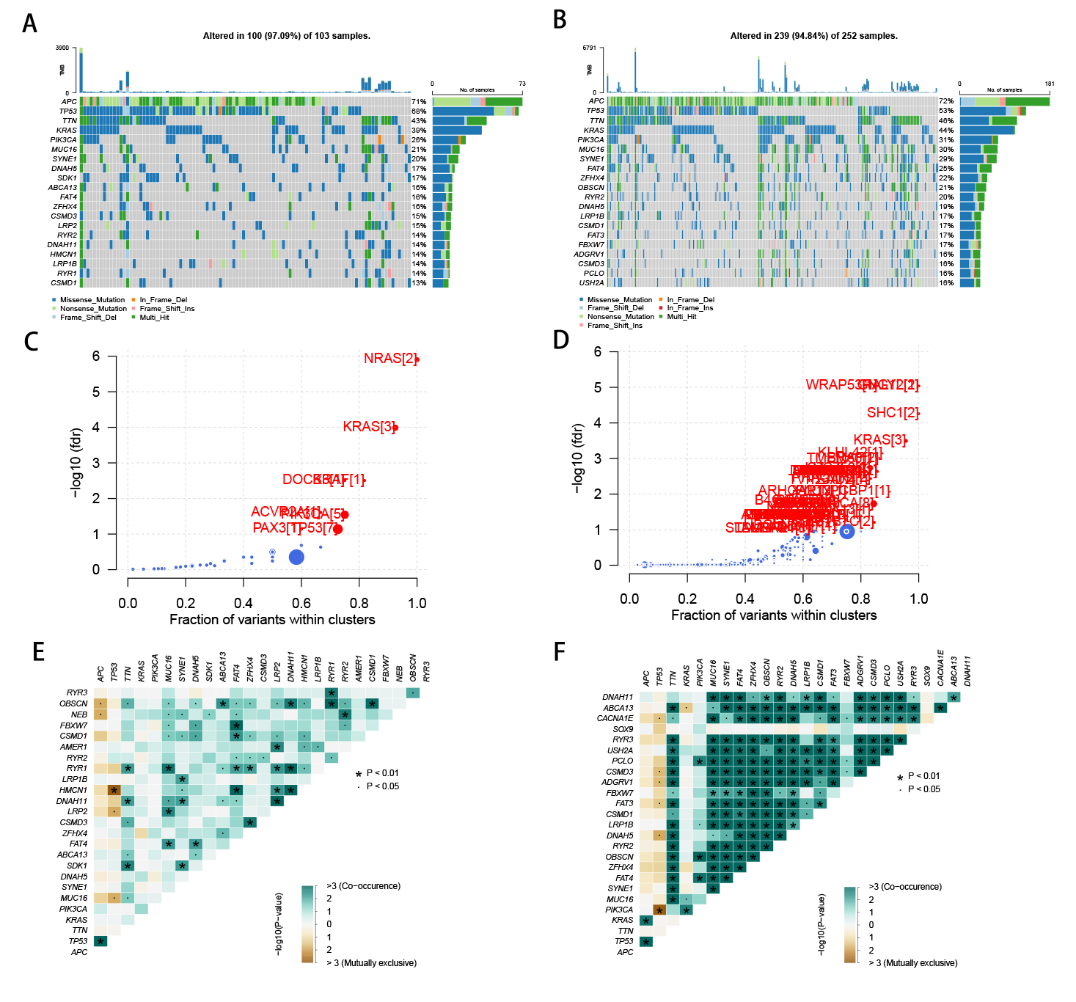


**Figure S4.** Somatic mutations alterations of related to the ARGs-based signature in CRC. The maftool exhibited the incidence of somatic mutations of ARGs in low- **(A)** and high-risk patients **(B)** of CRC. **C, D**. Identification of tumour driver genes in both cohorts. Comparison of co-occurrence and mutually exclusive mutations of the mutated genes between the high-risk **(E)** and low-risk groups **(F)**.


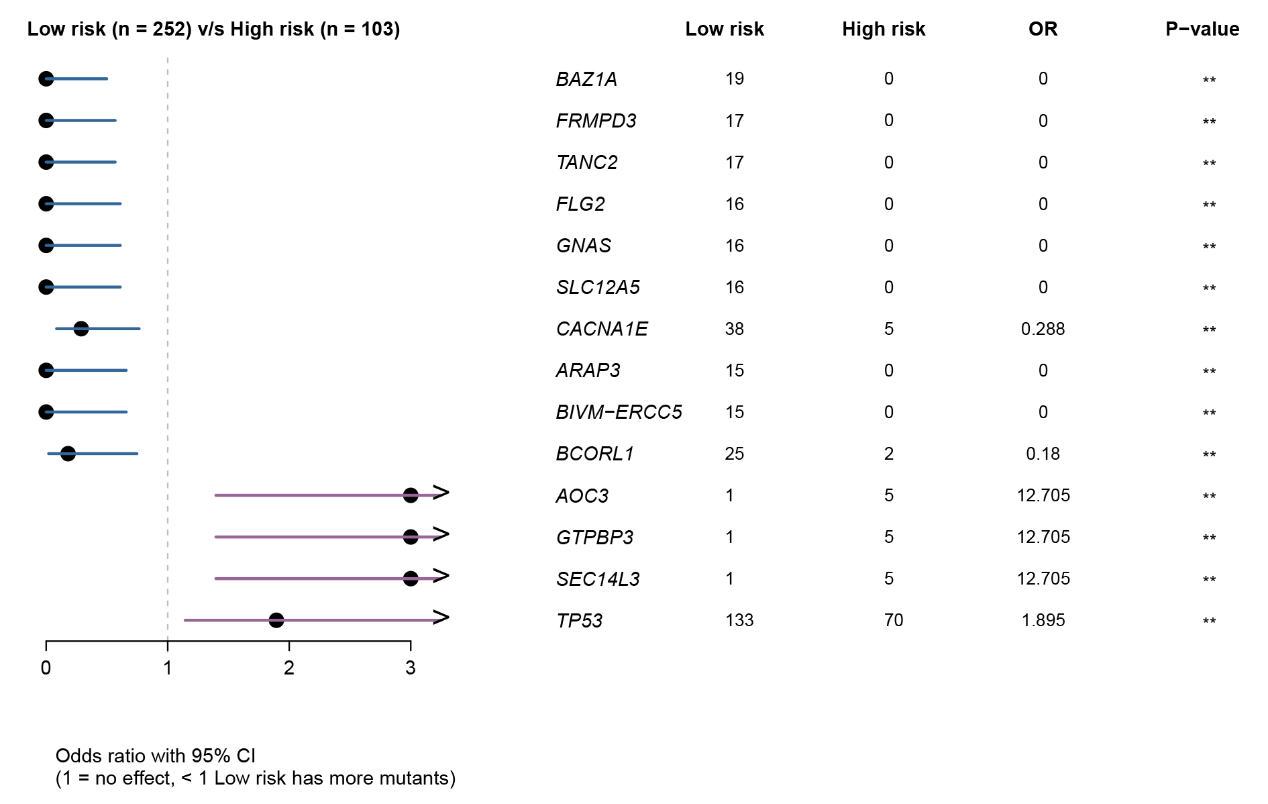


**Figure S5.** Forest plots showing the results of differentially mutated genes between the high and low-risk cohorts.


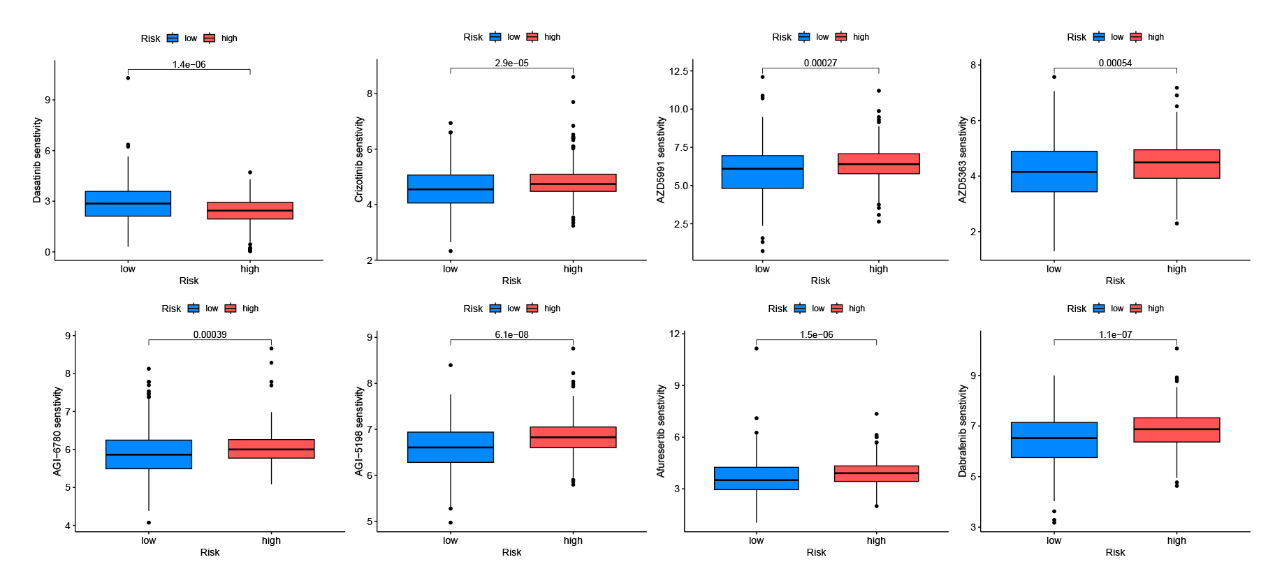


**Figure S6.** Screening of small-molecule drugs between high and low-risk patient subgroups


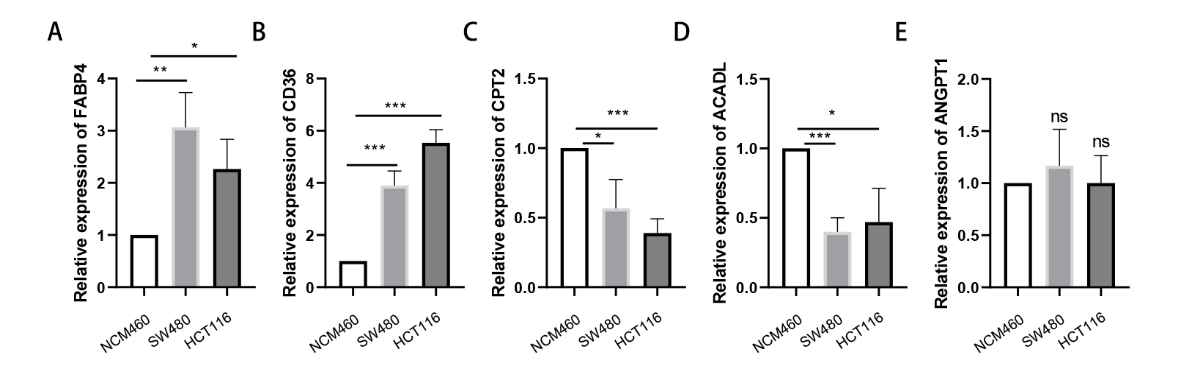


**Figure** **S7.** mRNA expression levels of ARGs in CRC cell line and normal cell line. RT-PCR results of FABP4 **(A)**, CD36 **(B)**, CPT2 **(C)**, ACADL **(D)** and ANGPT1 **(E)**.

The list of gene primers

ACADL Forward, 5'-TTGGCAAAACAGTTGCTCAC-3'. Reverse, 5'-ACATGTATCCCCAACCTCCA-3'. GAPDH Forward, 5'-GGACCTGACCTGCCGTCTAG-3’. Reverse, 5'-GTAGCCCAGGATGCCCTTGA-3'. Cd36 Forward: 5'-CTTTGGCTTAATGAGACTGGGAC-3' Reverse: 5'-GCAACAAACATCACCACACCA-3'. FABP4 Forward: 5'-TGGGCCAGGAATTTGACGA-3' Reverse: 5'-CATTTCTGCACATGTACCAGGACAC-3'. ANGPT1 Forward: 5'- TGCAGCAACCAGCGCCGAAA-3' Reverse: 5'-CAGGGCAGTTCCCGTCGTGT-3'. CPT2 Forward: 5'-AAAGAAGCAGCAATGGGCCAG-3' Reverse: 5'-CCAGGTAGAGCTCAGGCAAG-3'.
